# Supplementary material for: From sequence to enzyme mechanism using multi-label machine learning
Source: BMC Bioinformatics. 2014 May 19;15:150. doi: 10.1186/1471-2105-15-150 (PMC4229970; doi:10.1186/1471-2105-15-150)
Supplement: Additional file 2 — Java code of ml2db. Additional file ml2db_code.tar.gz contains the Java source code to run the multi-label machine learning experiments and save the results to database. The code’s Javadoc is included. [file 1471-2105-15-150-S2.zip › additional file 2/ml2db/ml2db/doc/main/ml2db/al/utility/pickers/byfrequency/AttributesByFrequencyPicker.html]

AttributesByFrequencyPicker


JavaScript is disabled on your browser.


- Overview
- Package
- Class
- Use
- Tree
- Deprecated
- Index
- Help

- Prev Class
- Next Class

- Frames
- No Frames

- All Classes

- Summary:
- Nested |
- Field |
- Constr |
- Method

- Detail:
- Field |
- Constr |
- Method


main.ml2db.al.utility.pickers.byfrequency

## Class AttributesByFrequencyPicker

- java.lang.Object
- - main.ml2db.al.utility.UtilityMetric
  - - main.ml2db.al.utility.pickers.RandomPicker
    - - main.ml2db.al.utility.pickers.byfrequency.ByFrequencyPicker
      - - main.ml2db.al.utility.pickers.byfrequency.AttributesByFrequencyPicker

- All Implemented Interfaces:
  :   Picker

  ---

    

  ```
  public class AttributesByFrequencyPicker
  extends ByFrequencyPicker
  ```

  Class

  Version:
  :   9 May 2012

  Author:
  :   Luna De Ferrari ldf(at)st-andrews.ac.uk or
      luna.deferrari@st-andrews.ac.uk

- - ### Field Summary

    - ### Fields inherited from class main.ml2db.al.utility.pickers.RandomPicker

      `m_debug, m_test`
  - ### Constructor Summary

    Constructors

    Constructor and Description

    `AttributesByFrequencyPicker()`
  - ### Method Summary

    Methods

    | Modifier and Type | Method and Description |
    | --- | --- |
    | `java.lang.String` | `getMetricName()` |

    - ### Methods inherited from class main.ml2db.al.utility.pickers.byfrequency.ByFrequencyPicker

      `calculateFrequency, getFeaturesByDescendentFrequency, getFrequencyToFeatureMap, initialise, orderAttsetByDescFreq, setFeaturesByDescendentFrequency, setFrequencyToFeatureMap`
    - ### Methods inherited from class main.ml2db.al.utility.pickers.RandomPicker

      `getInstanceToFeaturesMap, getQueries, getQuery, getStep, getUtility, isDebug, isTest, refresh, setDebug, setFeatureToInstanceMap, setStep, setTest`
    - ### Methods inherited from class main.ml2db.al.utility.UtilityMetric

      `getUnlabelled, setUnlabelled`
    - ### Methods inherited from class java.lang.Object

      `equals, getClass, hashCode, notify, notifyAll, toString, wait, wait, wait`

- - ### Constructor Detail


    - #### AttributesByFrequencyPicker

      ```
      public AttributesByFrequencyPicker()
      ```
  - ### Method Detail


    - #### getMetricName

      ```
      public java.lang.String getMetricName()
      ```

      **Specified by:**
      :   `getMetricName` in class `ByFrequencyPicker`


- Overview
- Package
- Class
- Use
- Tree
- Deprecated
- Index
- Help

- Prev Class
- Next Class

- Frames
- No Frames

- All Classes

- Summary:
- Nested |
- Field |
- Constr |
- Method

- Detail:
- Field |
- Constr |
- Method
